# Draft Genome Sequence of *Clostridium butyricum* Strain NOR 33234, Isolated from an Elderly Patient with Diarrhea

**DOI:** 10.1128/genomeA.01356-14

**Published:** 2014-12-24

**Authors:** Jamie S. L. Kwok, Margaret Ip, Ting-Fung Chan, Wai-Yip Lam, Stephen K. W. Tsui

**Affiliations:** a School of Biomedical Sciences, The Chinese University of Hong Kong, Hong Kong, China; b Department of Microbiology, The Chinese University of Hong Kong, Hong Kong, China; c School of Life Sciences, The Chinese University of Hong Kong, Hong Kong, China; d Centre for Microbial Genomics and Proteomics, The Chinese University of Hong Kong, Hong Kong, China

## Abstract

*Clostridium butyricum* is one of the species frequently present in patients’ stool samples. However, the identification of this species is sometimes difficult. Here, we present the draft genome of *Clostridium butyricum* NOR 33234, which was isolated from a patient with suspected *Clostridium difficile* infection-associated diarrhea and resembles *Clostridium clostridioforme* in biochemical tests.

## GENOME ANNOUNCEMENT

The varied roles of *Clostridium butyricum* range from a promising biofuel producer (1) to a neurotoxigenic pathogen (2). Toxin-free strains are used in probiotics, especially in preventing *Clostridium diffiicle*-related diarrhea (3). Recently, the genome of strain 5521 carrying botulinum neurotoxin type E (*boNT/E*) has been sequenced (4).

A stool sample from a 94-year-old male patient with suspected *C. diffiicle* infection (CDI)-associated diarrhea was collected. A strain was isolated and identified by VITEK2 as *Clostridium clostridioforme*. Among comprehensive biochemical tests, only α- and β-galactosidase activities were positive (^+^) (AGALi^+^, BGALi^+^). Previous reports suggest that besides *C. difficile*, *Clostridium perfringens* (5, 6), or other *Clostridium* spp. may also cause antibiotic-associated diarrhea, of which CDI is the major cause. To understand the nature of the strain, we sequenced the genome using Ion Torrent single-end libraries, of which 6,595,984 reads were assembled into 70 long contigs (>10,000 bp) using MIRA (version 4.0.2) (7) and gap5 (8), with an *N*_50_ of 156,811 bp. In contrast to biochemical identification, 16S analysis using 16SpathDB 2.0 (9) identified our sample as *C. butyricum* with 99.0% nucleotide identity, within the species definition threshold. The GC content of the genome is 28.37%, which is also similar to that of published *C. butyricum* genomes and very different from that of *C. clostridioforme* (GC content, ~49%).

Protein prediction is done using the NCBI prokaryotic genome annotation pipeline (10), resulting in 4,357 genes, with 4,085 coding genes and 272 pseudogenes. When all the coding genes were searched against nonredundant protein databases in NCBI, 3,901 genes had significant hits (*E* value ≤1e^-30^). Among them, 3,714 (95.21%) have top hits to *C. butyricum*. Among these proteins, there is an annotated enterotoxin (OA81_00270), which has the highest homology (99.6% identity) to an SH3/3D protein (WP_024041145.1) in a *C. butyricum* strain isolated from human gut (11). Moreover, two annotated proteins (OA81_15790 and OA81_07195) have sequence similarity to phage holins (WP_003370185.1 and WP_024040534.1) in *C. botulinum*. A previous report has shown that holin-like *tcdE* is required for exporting enterotoxins *tcdA/B* in *C. difficile* (12). Whether the enterotoxin or holin plays a pathogenic role in *C. diffiicle* infection remains to be examined. In conclusion, genome sequencing is a more accurate method for the identification of *C. butyricum.*

### Nucleotide sequence accession numbers.

This Whole Genome Shotgun project has been deposited in GenBank under the accession no. JSEG00000000. The version described in this paper is the first version, JSEG01000000.
